# Supplementary figures and images for: Dynamic 1D search and processive nucleosome translocations by RSC and ISW2 chromatin remodelers
Source: eLife. 2024 Mar 18;12:RP91433. doi: 10.7554/eLife.91433 (PMC10948146; doi:10.7554/eLife.91433)

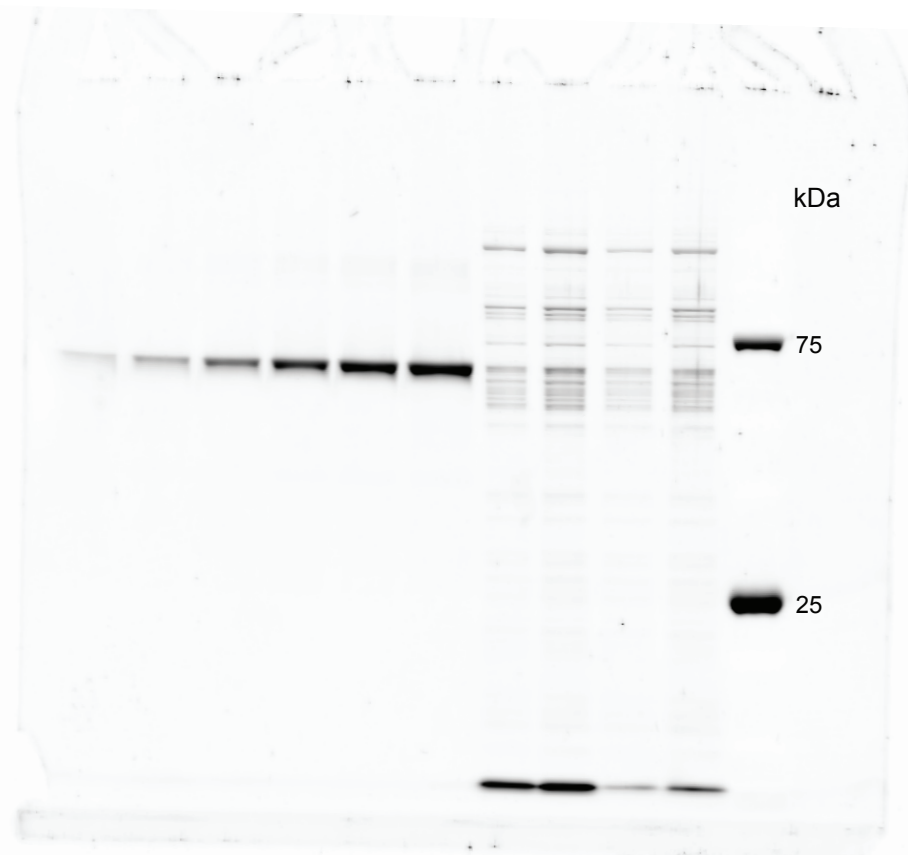

Supplement: Figure 1—figure supplement 1—source data 1. [file elife-91433-fig1-figsupp1-data1.pdf]

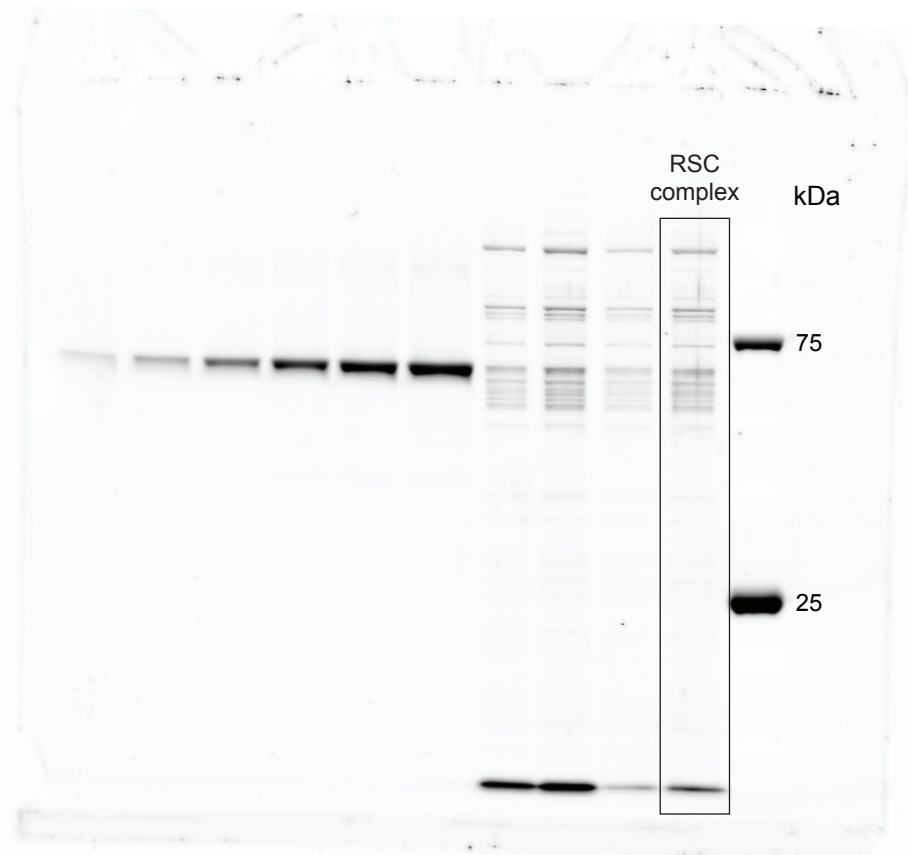

RSC  
complex

kDa

75

25

Supplement: Figure 1—figure supplement 1—source data 2. [file elife-91433-fig1-figsupp1-data2.pdf]

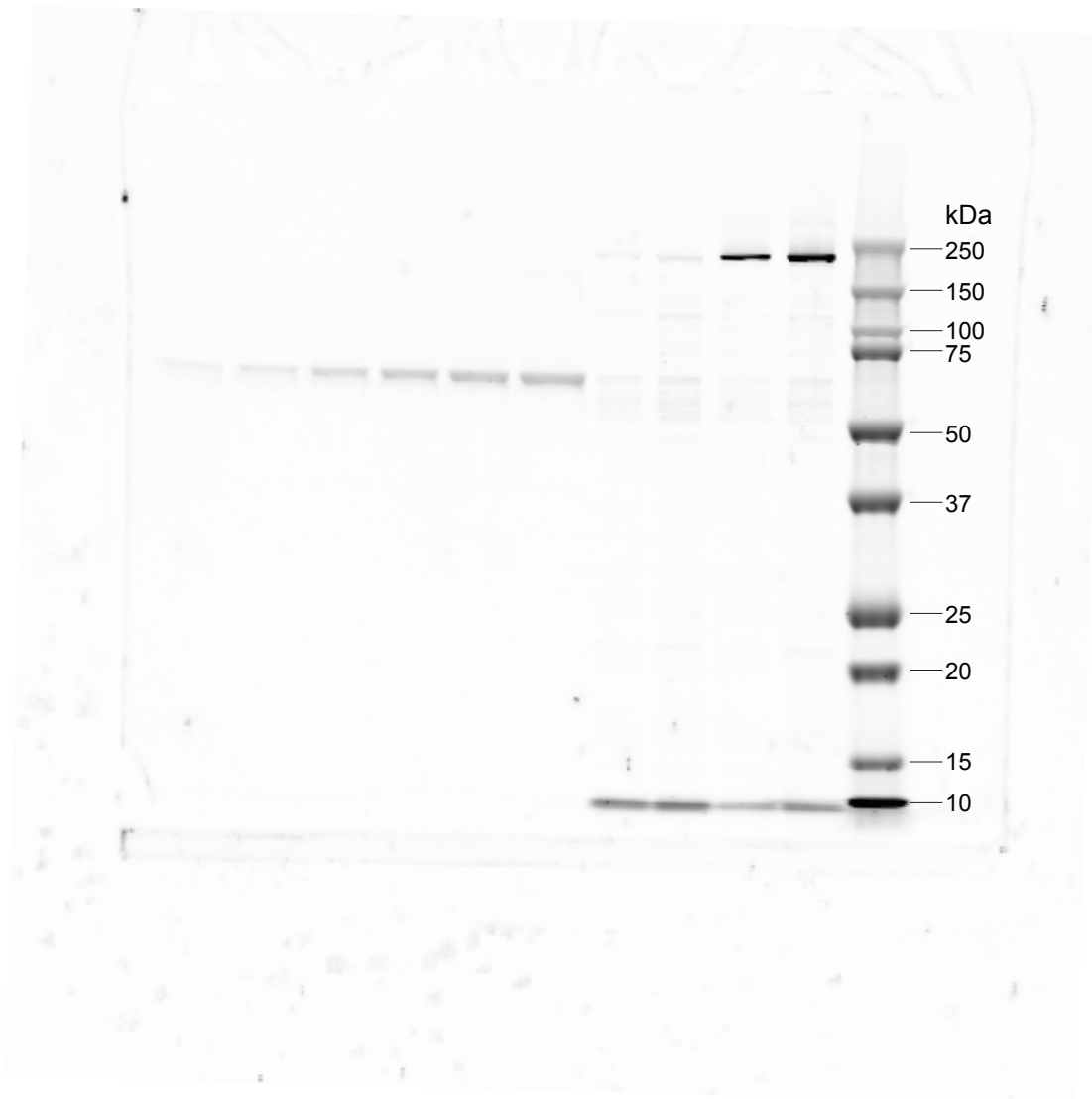

Supplement: Figure 1—figure supplement 1—source data 3. [file elife-91433-fig1-figsupp1-data3.pdf]

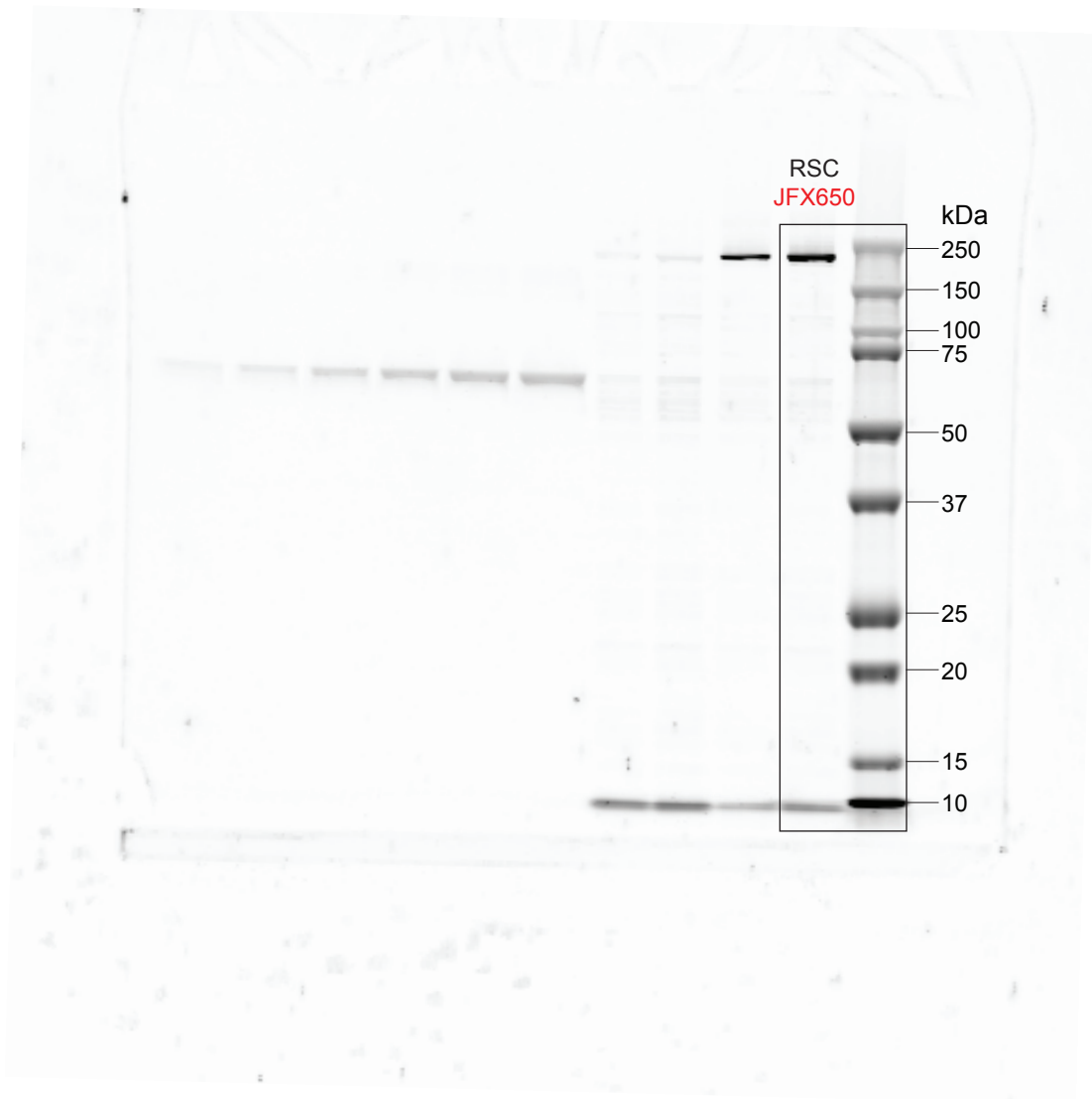

Supplement: Figure 1—figure supplement 1—source data 4. [file elife-91433-fig1-figsupp1-data4.pdf]

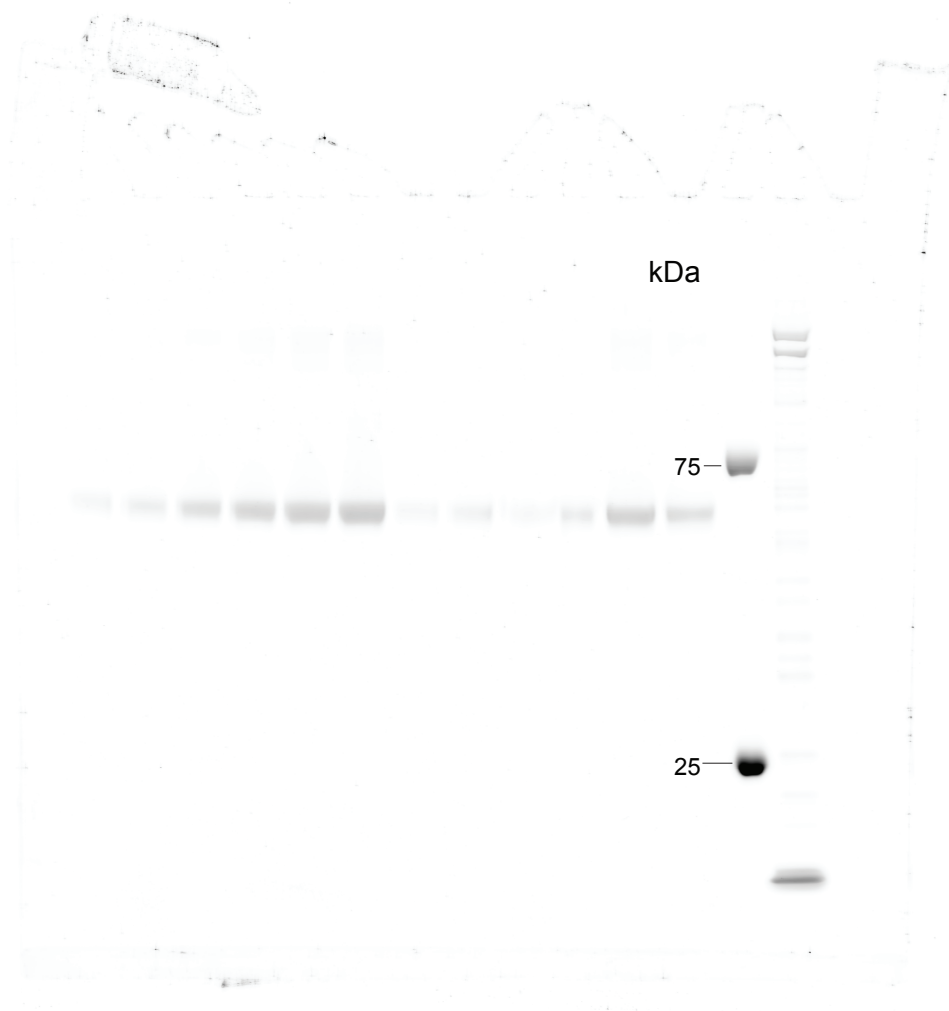

Supplement: Figure 1—figure supplement 1—source data 5. [file elife-91433-fig1-figsupp1-data5.pdf]

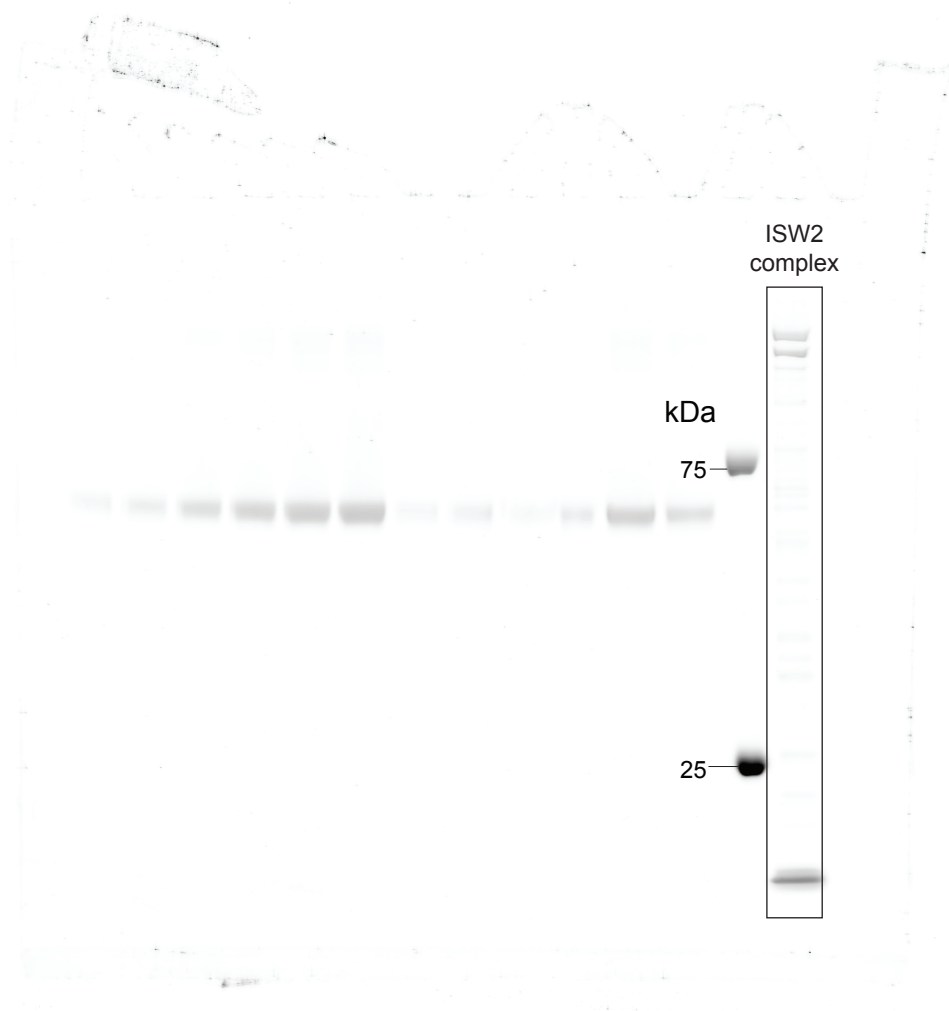

Supplement: Figure 1—figure supplement 1—source data 6. [file elife-91433-fig1-figsupp1-data6.pdf]

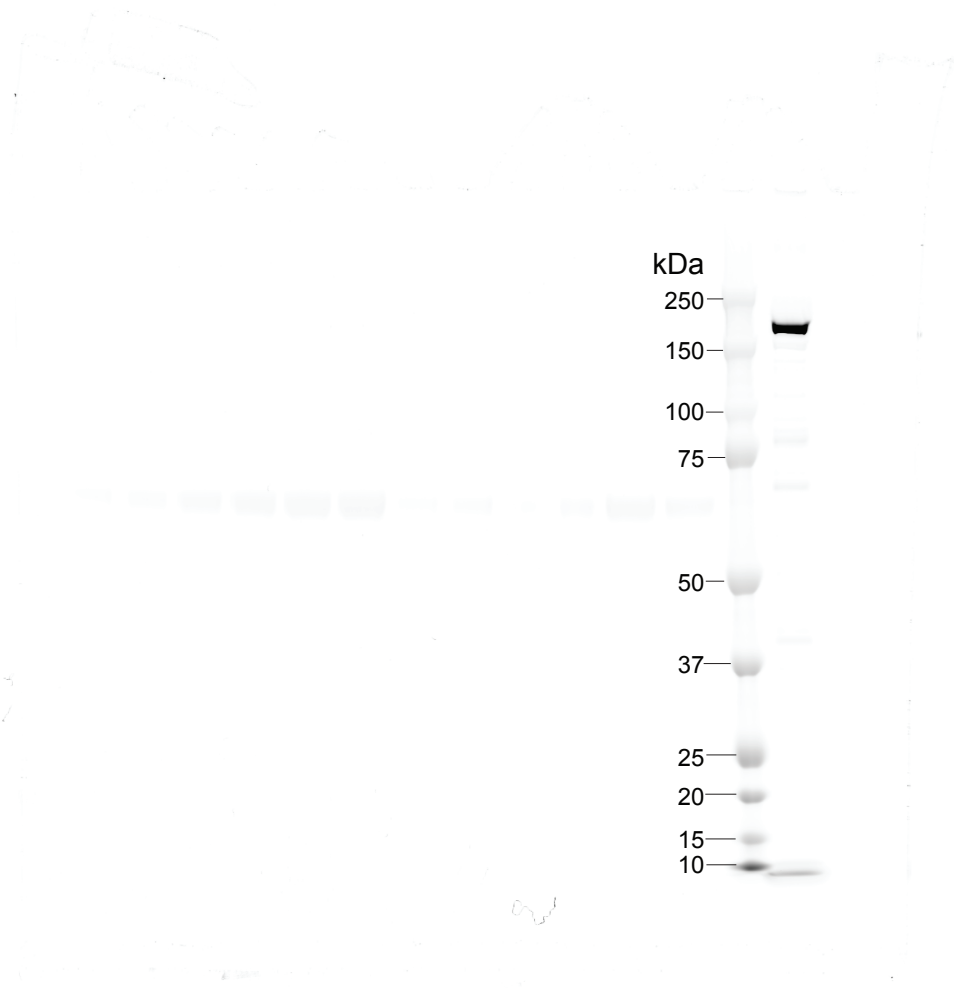

kDa

250—

150—

100—

75—

50—

37—

25—

20—

15—

10—

Supplement: Figure 1—figure supplement 1—source data 7. [file elife-91433-fig1-figsupp1-data7.pdf]

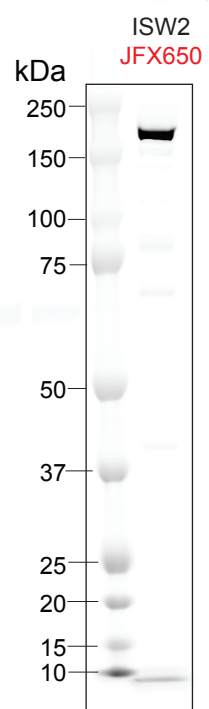

Supplement: Figure 1—figure supplement 1—source data 8. [file elife-91433-fig1-figsupp1-data8.pdf]

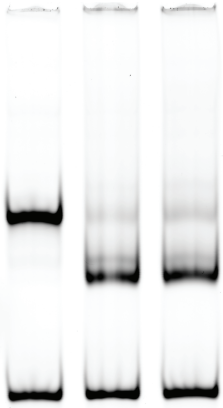

Supplement: Figure 1—figure supplement 2—source data 1. [file elife-91433-fig1-figsupp2-data1.zip › Fig1-figsupp2-SourceData1/Fig1-figsupp2-SourceData1.pdf]

RSC (Sth1-3F-Halo)

30°C

30"

15"

30"

—

+

+

1 mM ATP

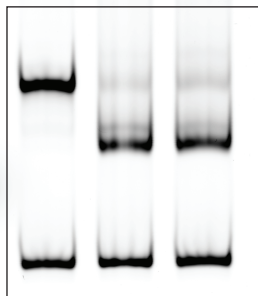

Supplement: Figure 1—figure supplement 2—source data 2. [file elife-91433-fig1-figsupp2-data2.zip › Fig1-figsupp2-SourceData2/Fig1-figsupp2-SourceData2.pdf]

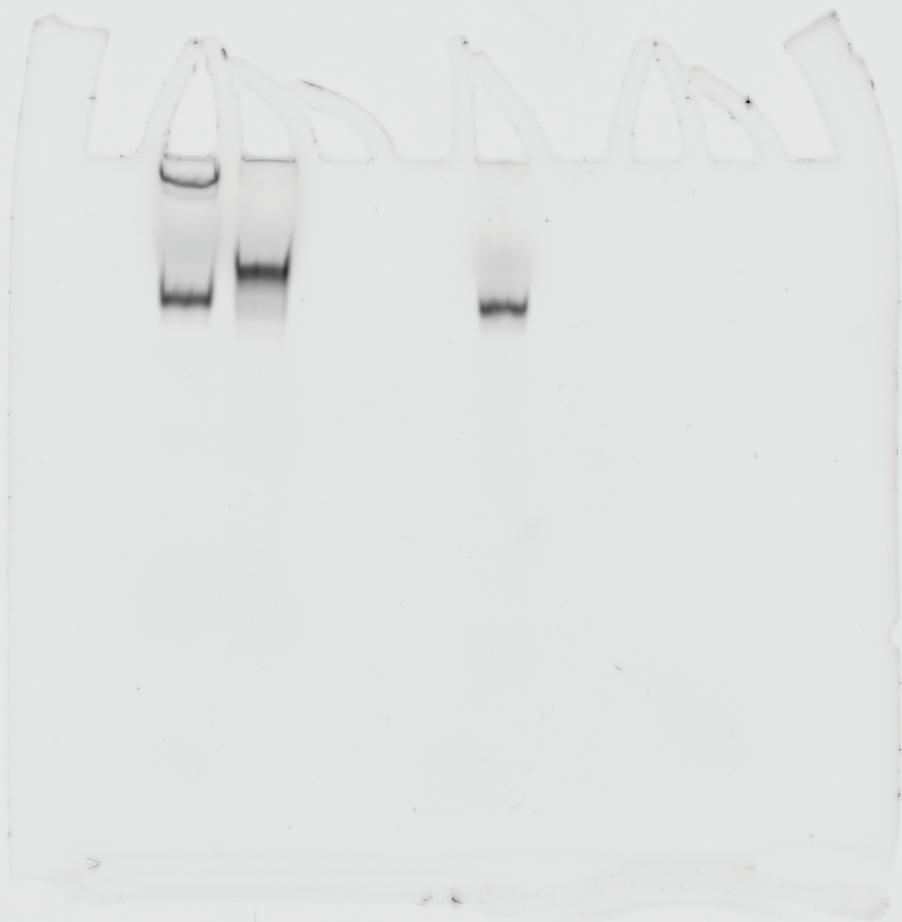

Supplement: Figure 1—figure supplement 2—source data 3. [file elife-91433-fig1-figsupp2-data3.zip › Fig1-figsupp2-SourceData3/Fig1-figsupp2-SourceData3.pdf]

ISW2 (Isw2-3F-Halo)  
30°C 30" 30"  
1 mM ATP — +  
80N3;  
Cy5-DNA  
Cy3-H2A

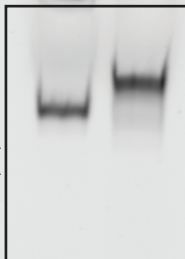

Supplement: Figure 1—figure supplement 2—source data 4. [file elife-91433-fig1-figsupp2-data4.zip › Fig1-figsupp2-SourceData4/Fig1-figsupp2-SourceData4.pdf]

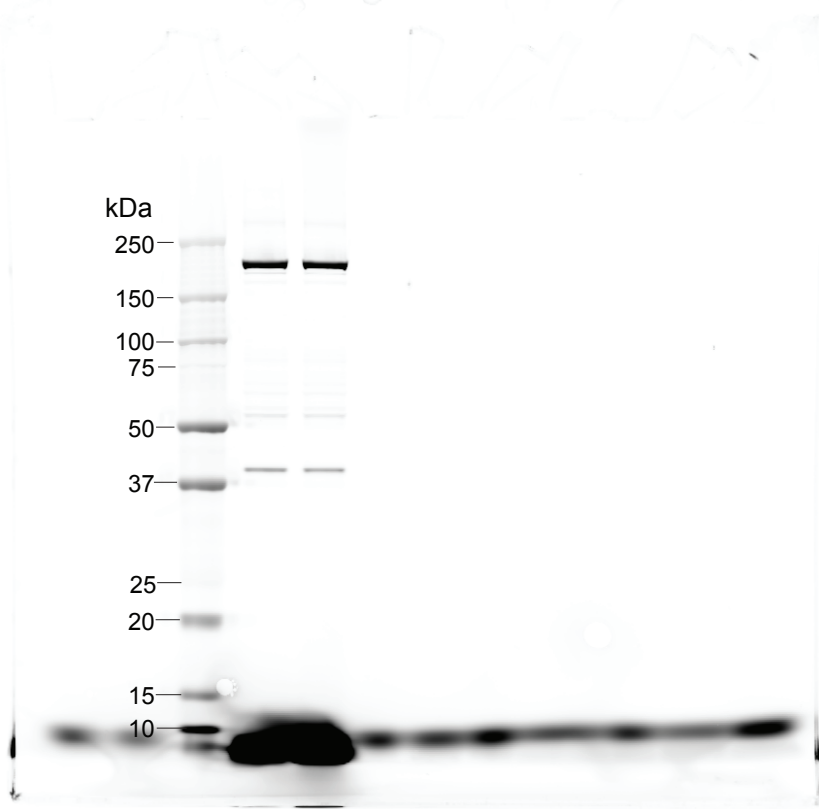

Supplement: Figure 1—figure supplement 2—source data 5. [file elife-91433-fig1-figsupp2-data5.pdf]

RSC labeling efficiency

**JFX650**

kDa      **2**    **5**     $\mu$ M

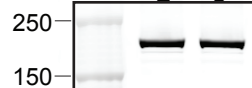

Cy5 channel  
excitation

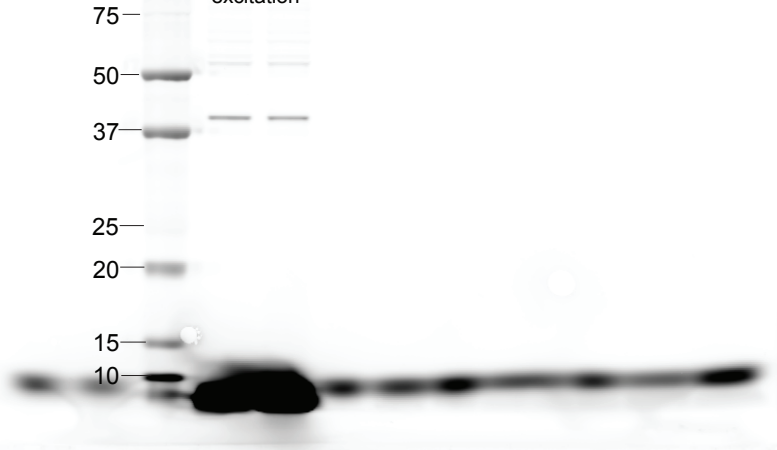

Supplement: Figure 1—figure supplement 2—source data 6. [file elife-91433-fig1-figsupp2-data6.pdf]

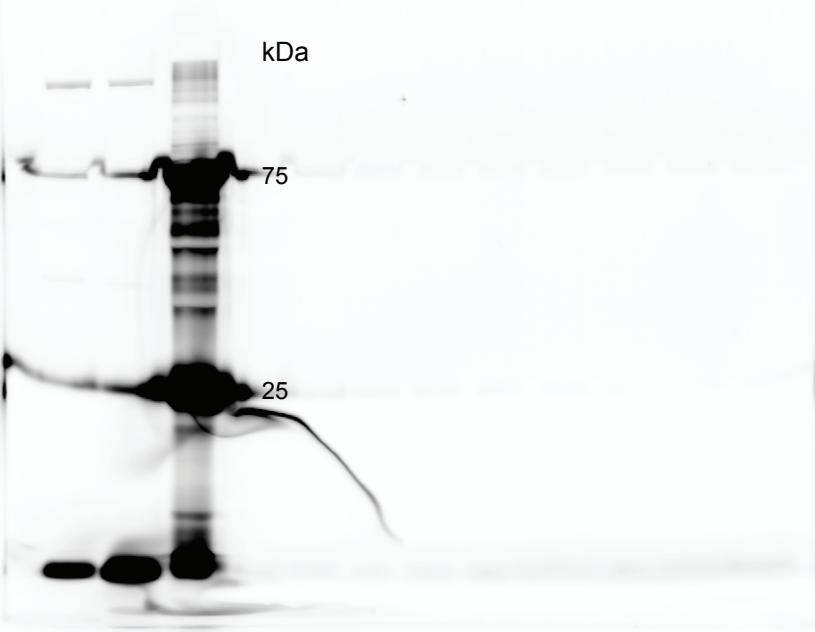

Supplement: Figure 1—figure supplement 2—source data 7. [file elife-91433-fig1-figsupp2-data7.pdf]

RSC labeling efficiency

JFX554

2 5  $\mu$ M

$\mu$ M

kDa

Cy3 channel  
excitation

75

25

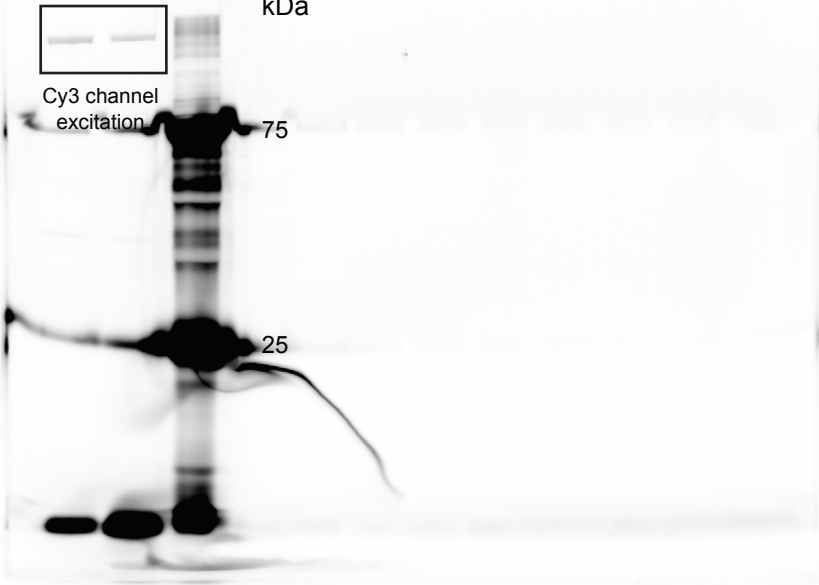

Supplement: Figure 1—figure supplement 2—source data 8. [file elife-91433-fig1-figsupp2-data8.pdf]

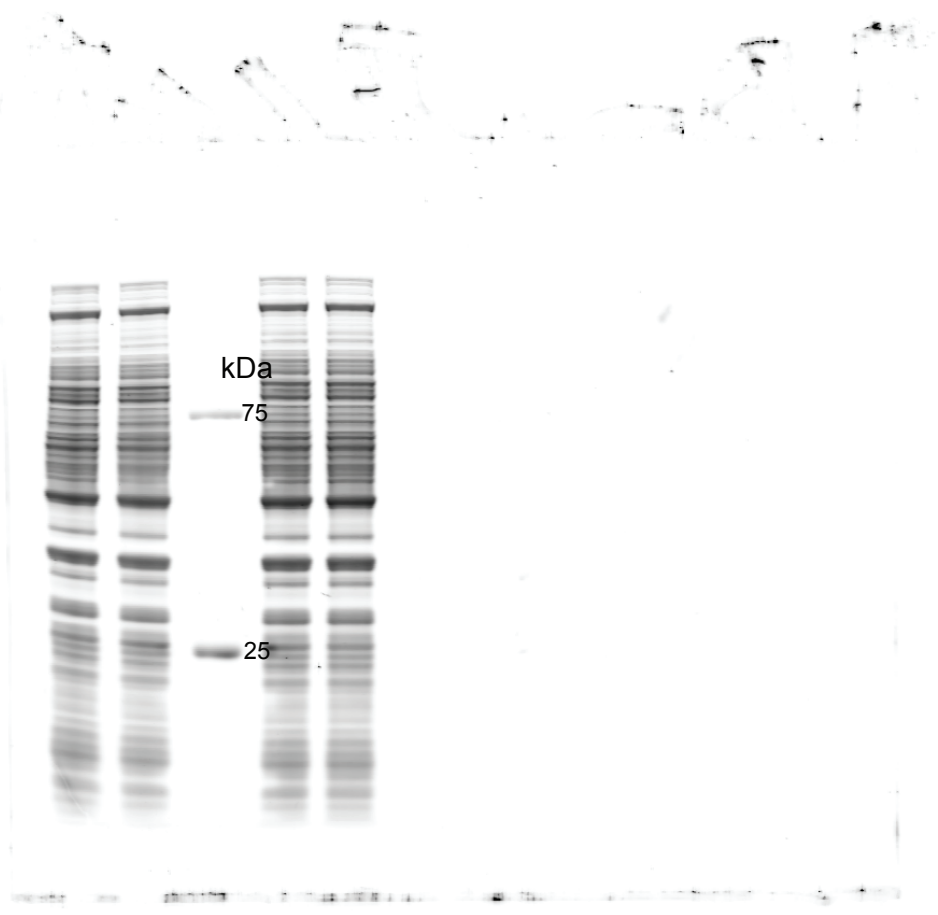

Supplement: Figure 1—figure supplement 2—source data 9. [file elife-91433-fig1-figsupp2-data9.pdf]

RSC labeling efficiency

**JFX554**

**JFX650**

2 5

2 5

$\mu\text{M}$

75

Flamingo stain - total protein

kDa

25

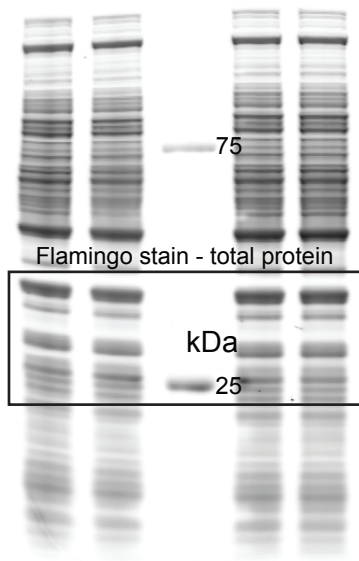

Supplement: Figure 1—figure supplement 2—source data 10. [file elife-91433-fig1-figsupp2-data10.pdf]

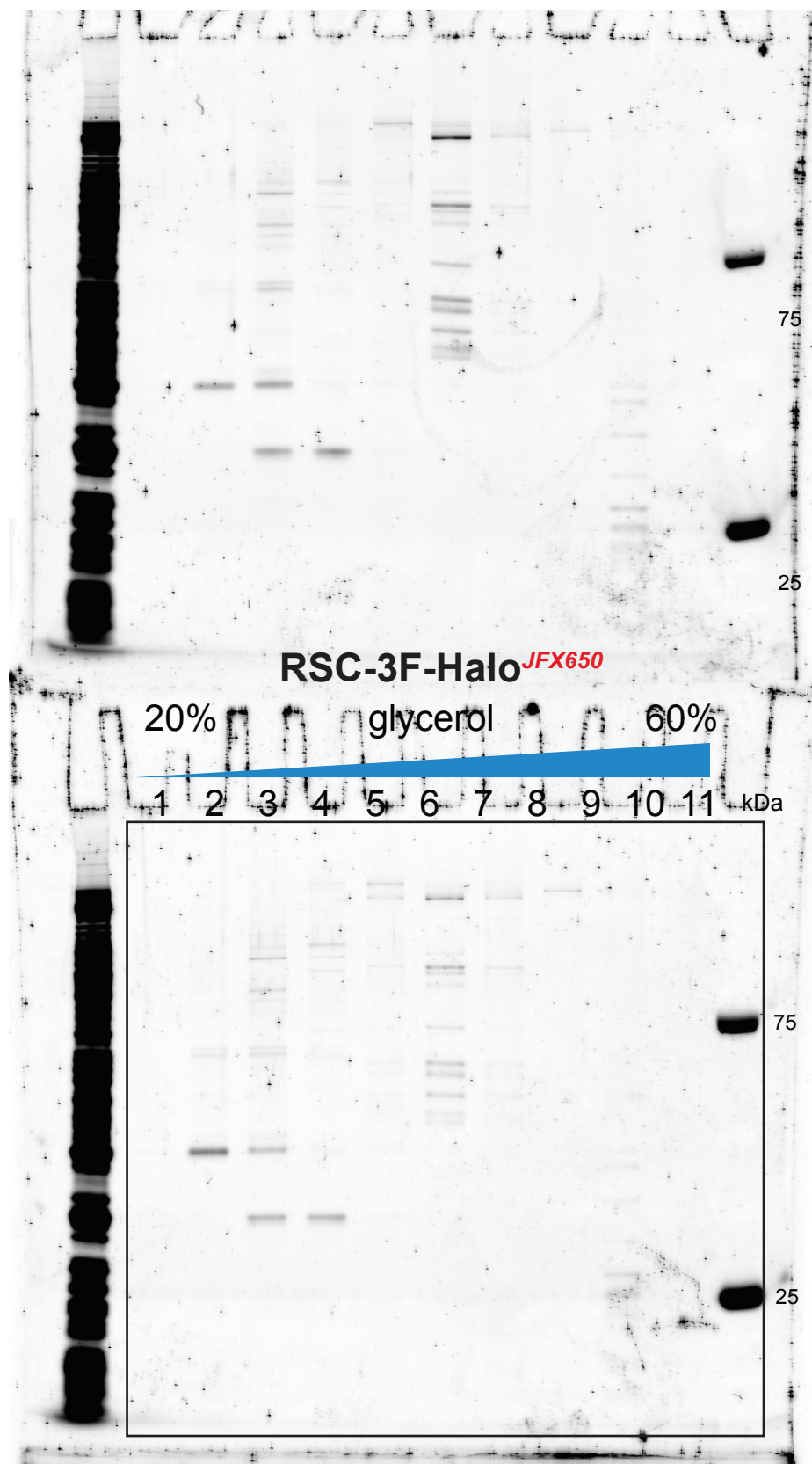

Flamingo stain channel excitation

Supplement: Figure 1—figure supplement 2—source data 12. [file elife-91433-fig1-figsupp2-data12.pdf]

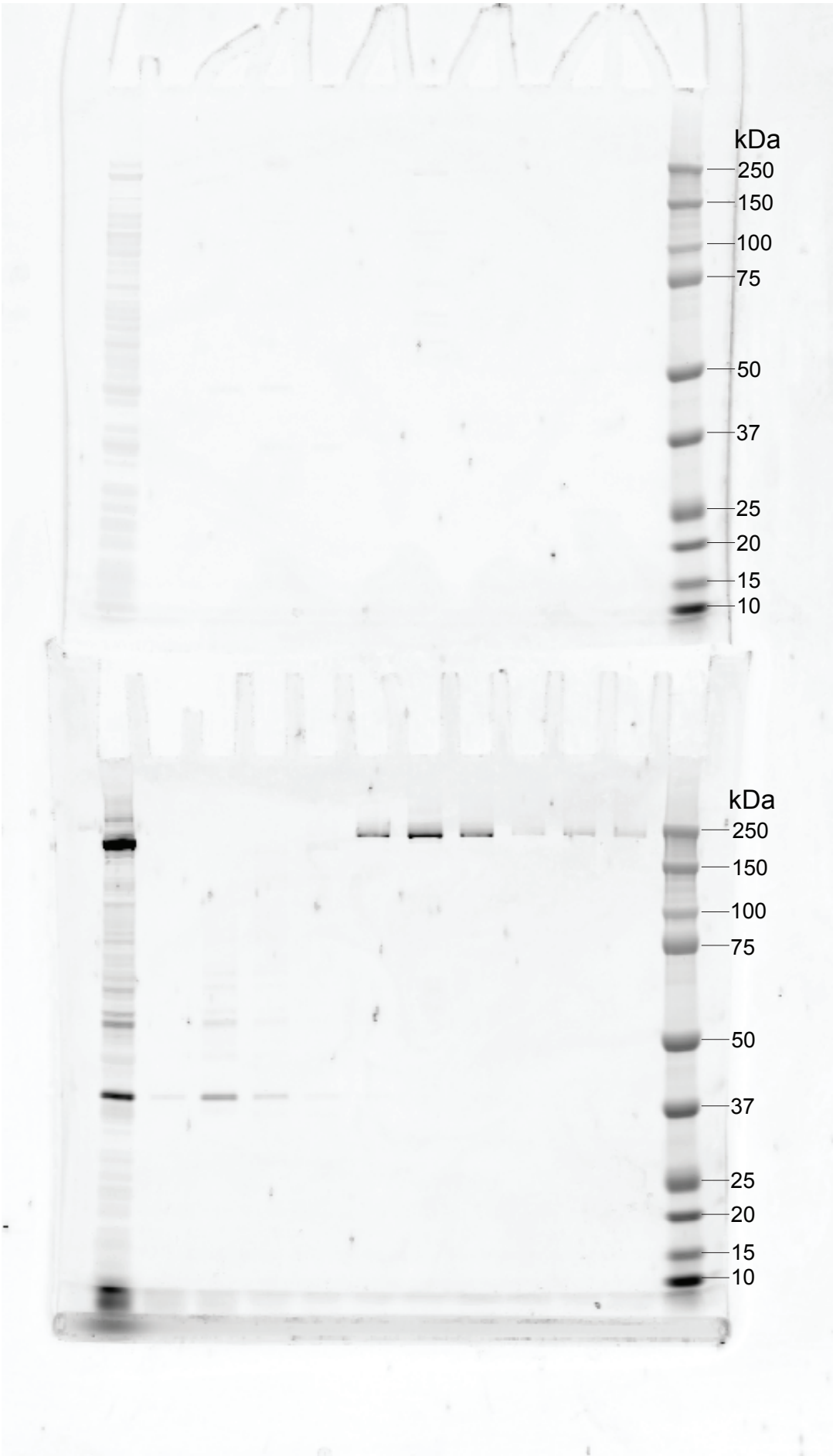

Supplement: Figure 1—figure supplement 2—source data 13. [file elife-91433-fig1-figsupp2-data13.pdf]

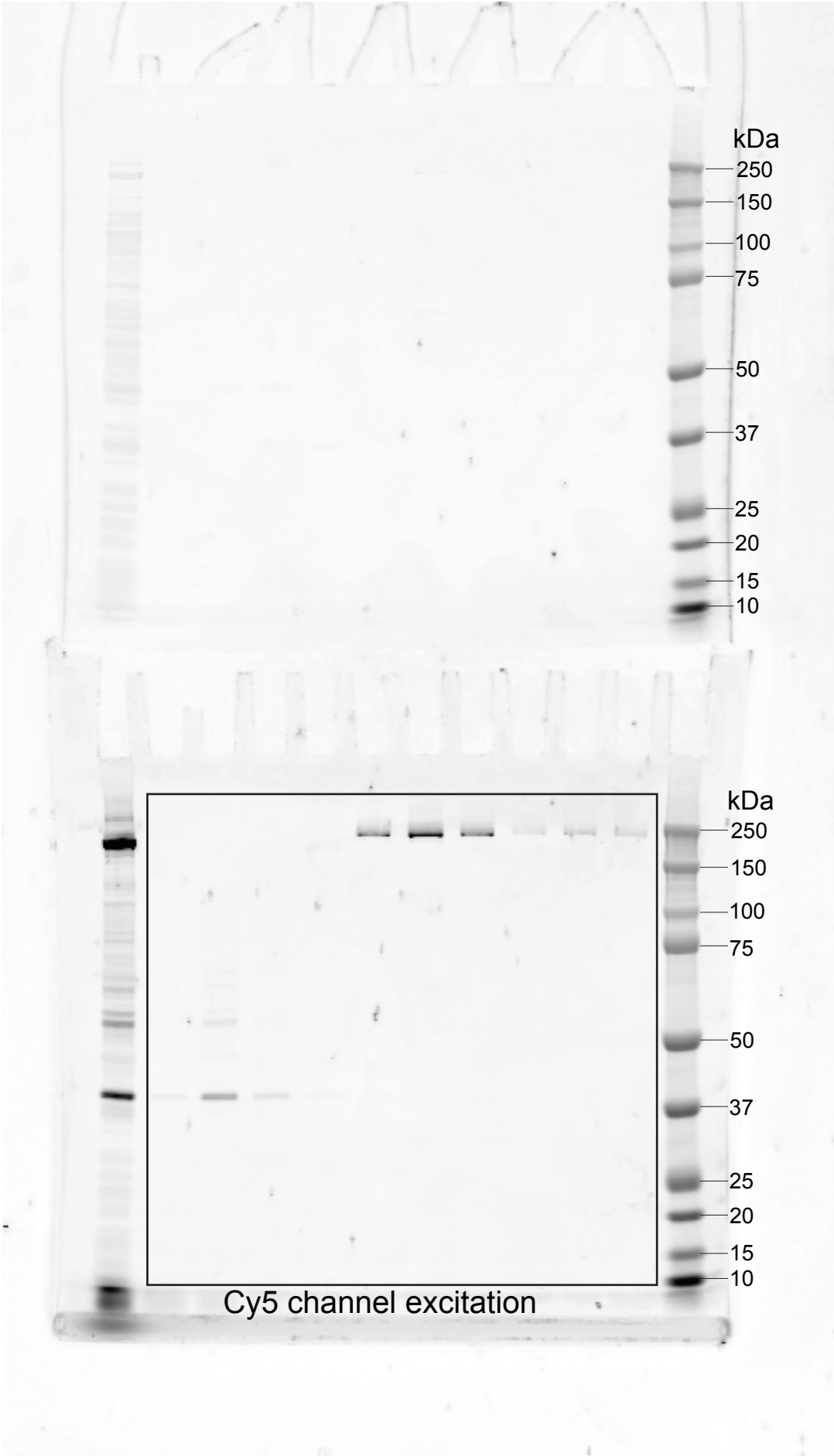

Supplement: Figure 1—figure supplement 2—source data 14. [file elife-91433-fig1-figsupp2-data14.pdf]

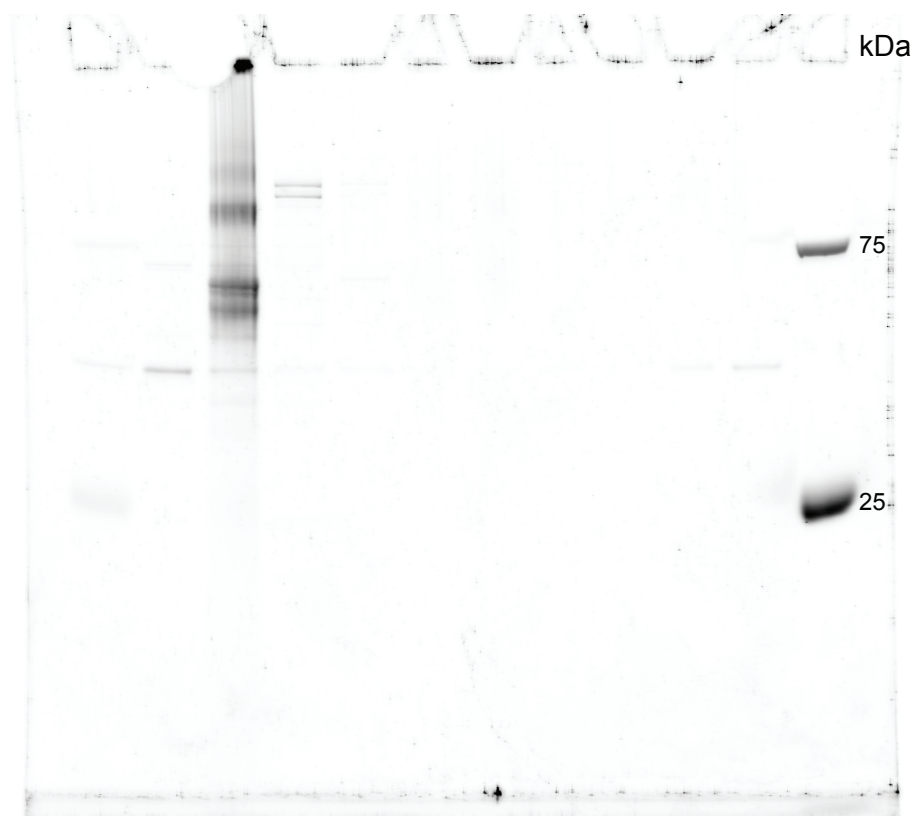

Supplement: Figure 1—figure supplement 2—source data 15. [file elife-91433-fig1-figsupp2-data15.pdf]

# ISW2-3F-Halo *JFX650*

20%

glycerol

60%

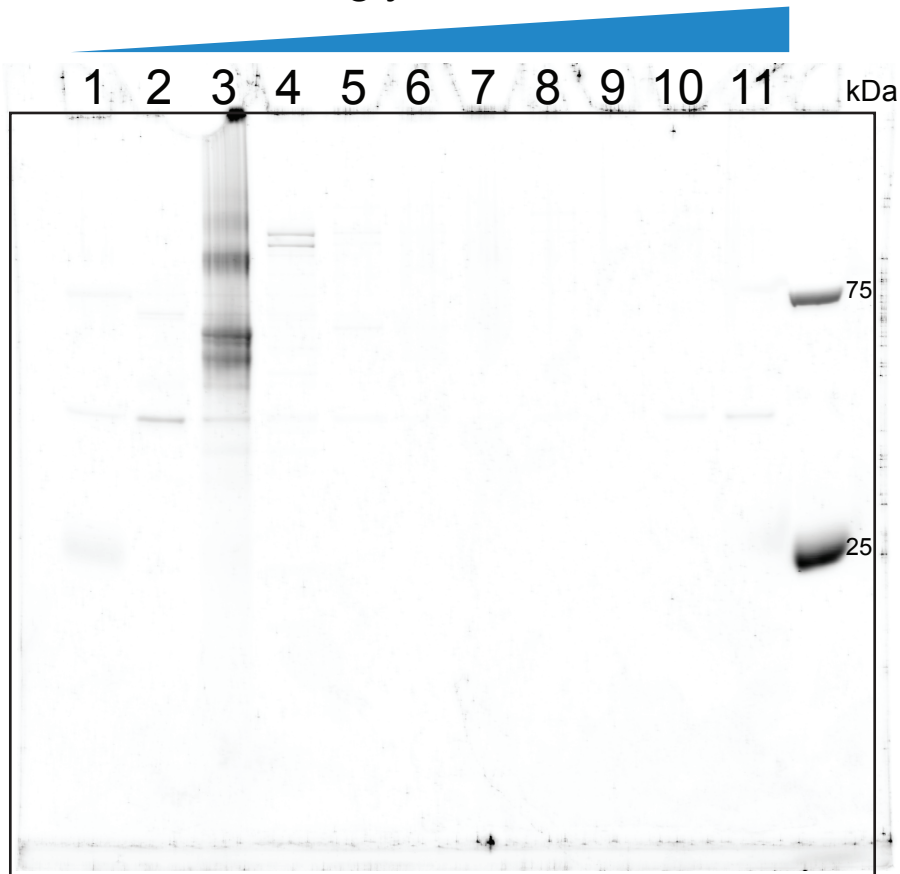

Flamingo stain channel excitation

Supplement: Figure 1—figure supplement 2—source data 16. [file elife-91433-fig1-figsupp2-data16.pdf]

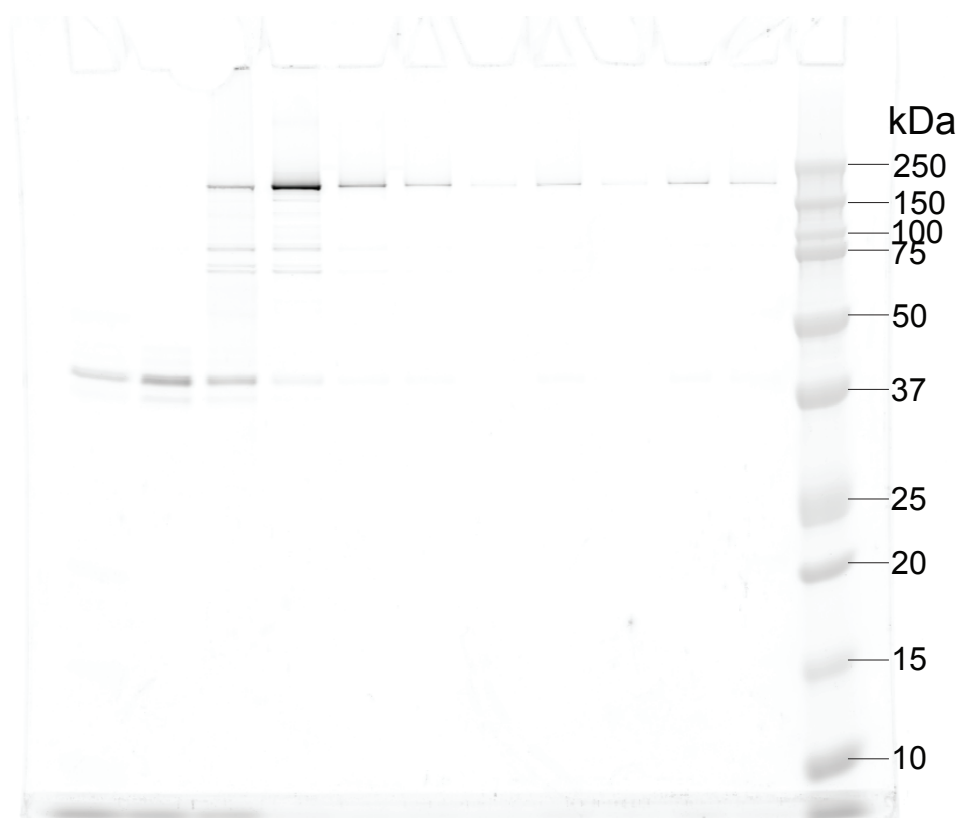

Supplement: Figure 1—figure supplement 2—source data 17. [file elife-91433-fig1-figsupp2-data17.pdf]

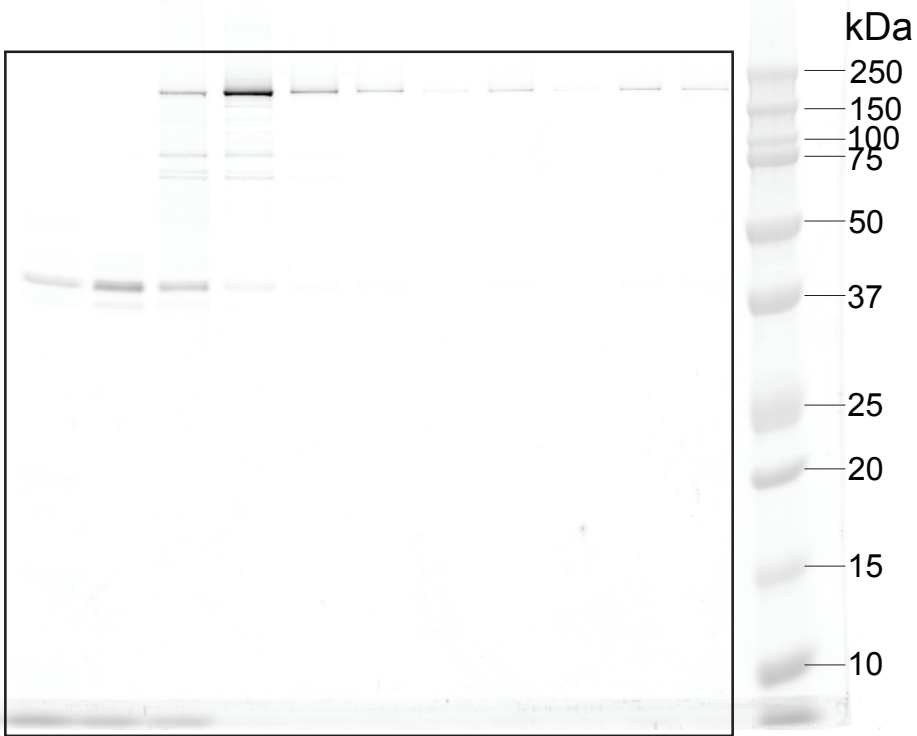

Cy5 channel excitation

Supplement: Figure 1—figure supplement 2—source data 18. [file elife-91433-fig1-figsupp2-data18.pdf]

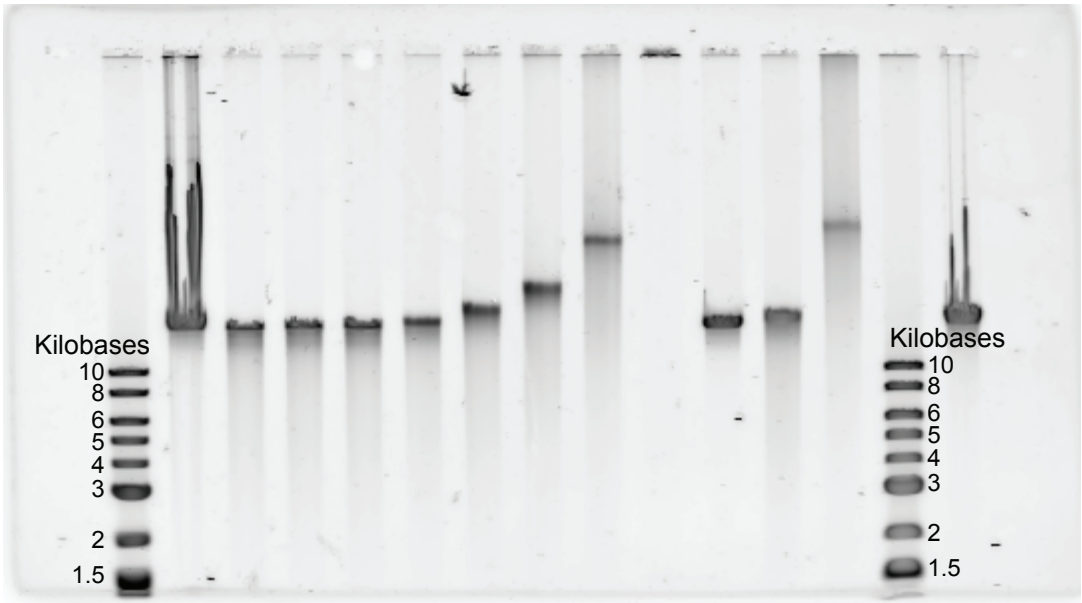

Supplement: Figure 3—figure supplement 1—source data 1. [file elife-91433-fig3-figsupp1-data1.pdf]

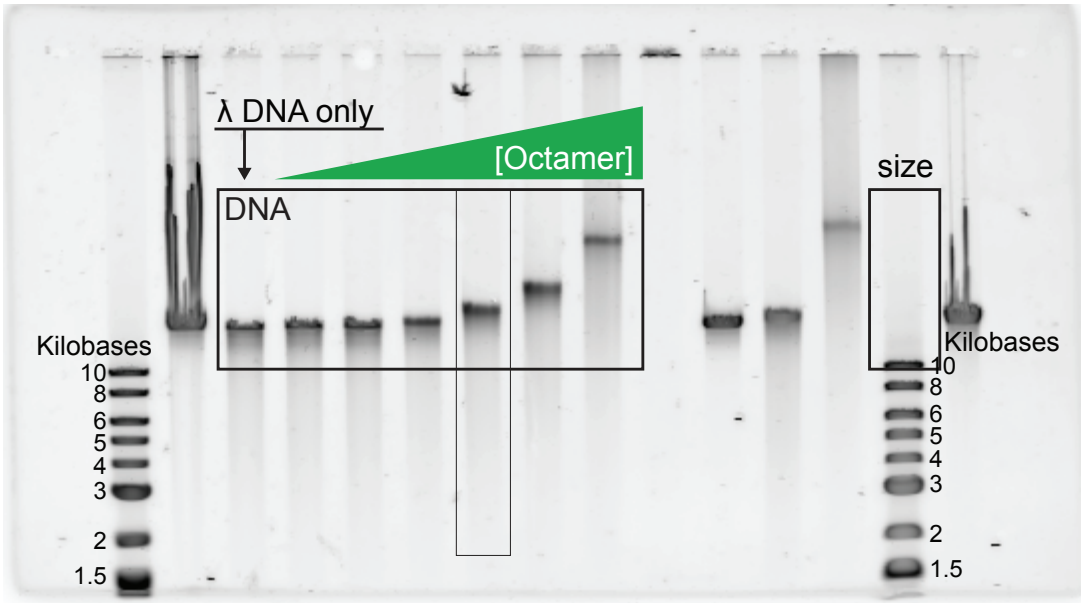

Supplement: Figure 3—figure supplement 1—source data 2. [file elife-91433-fig3-figsupp1-data2.pdf]

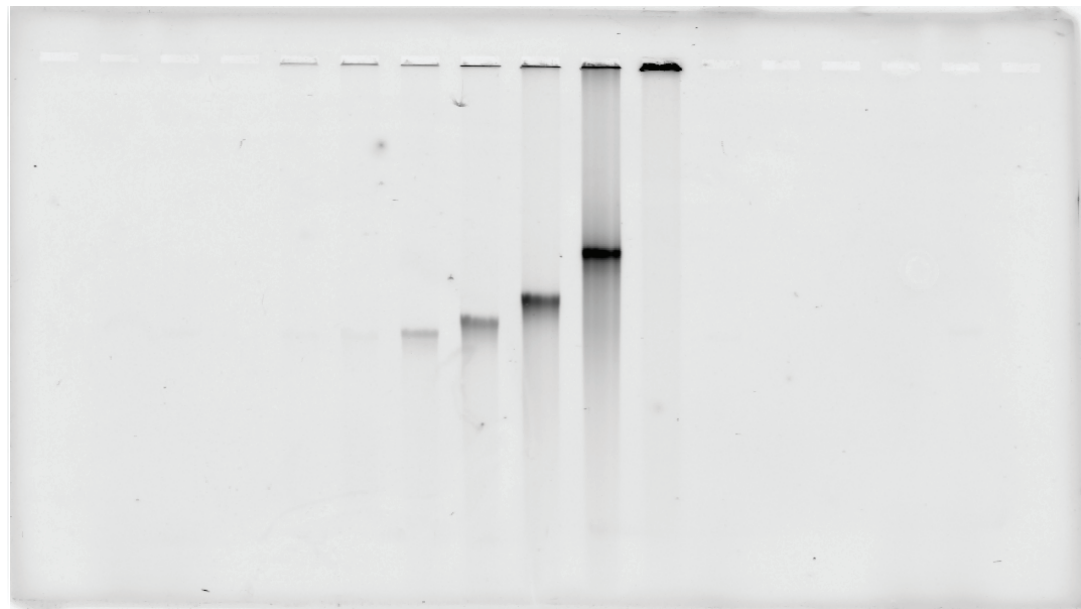

Supplement: Figure 3—figure supplement 1—source data 3. [file elife-91433-fig3-figsupp1-data3.zip › Fig3-figsupp1-SourceData3/Fig3-figsupp1-SourceData3.pdf]

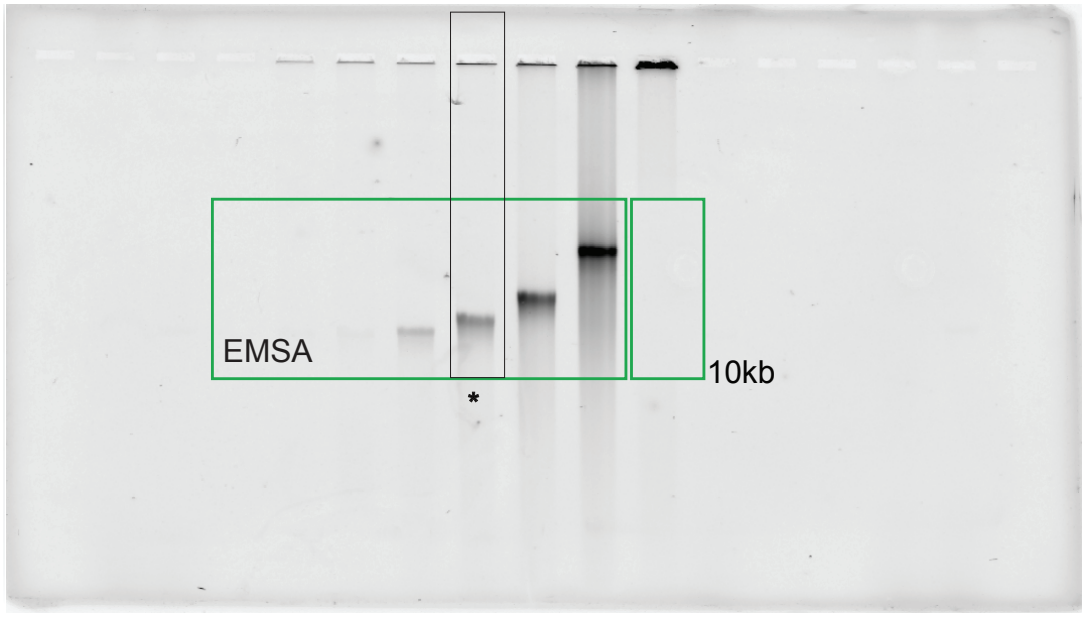

Supplement: Figure 3—figure supplement 1—source data 4. [file elife-91433-fig3-figsupp1-data4.zip › Fig3-figsupp1-SourceData4/Fig3-figsupp1-SourceData4.pdf]
