# Supplementary material for: Dynamic 1D search and processive nucleosome translocations by RSC and ISW2 chromatin remodelers
Source: eLife. 2024 Mar 18;12:RP91433. doi: 10.7554/eLife.91433 (PMC10948146; doi:10.7554/eLife.91433)
Supplement: Figure 1—figure supplement 2—source data 11. [file elife-91433-fig1-figsupp2-data11.pdf]

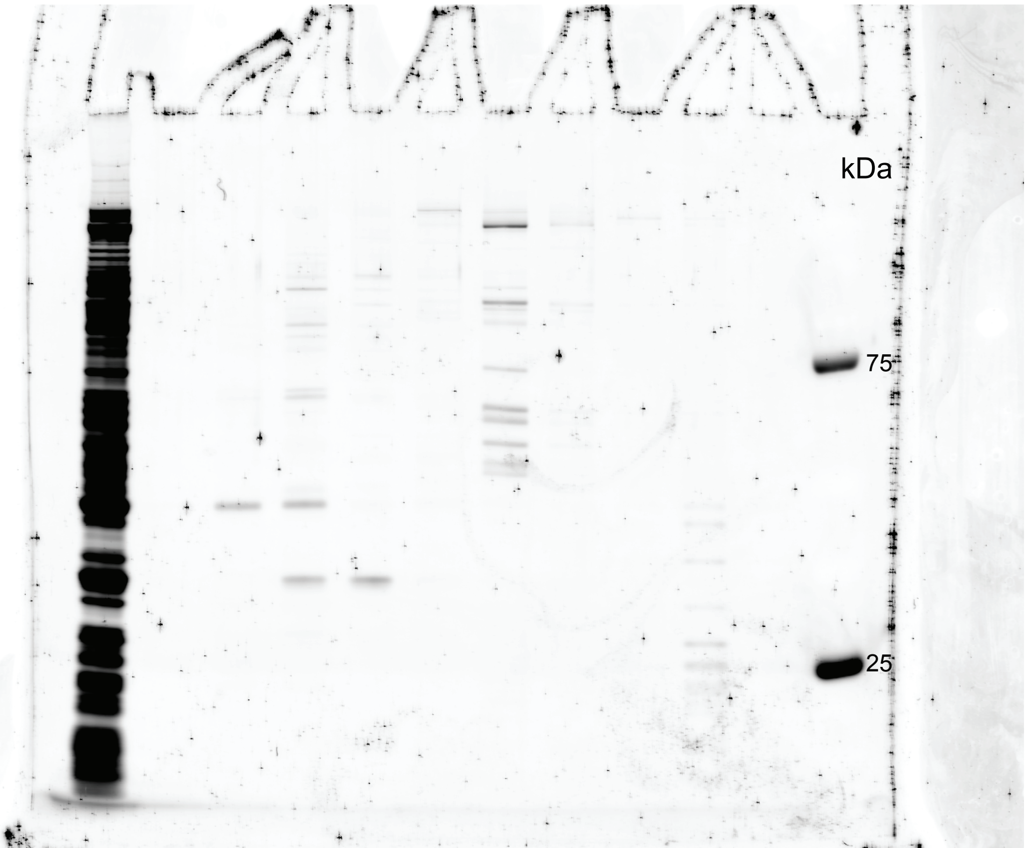
kDa
75
25

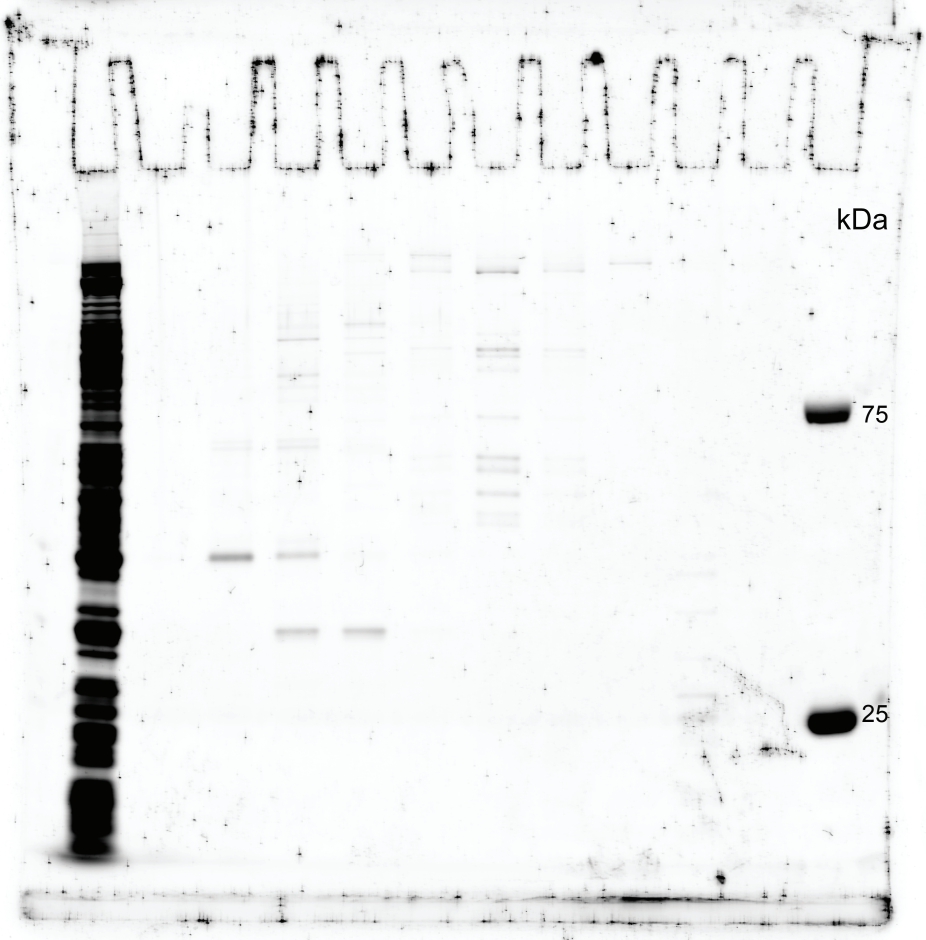
kDa
75
25
